# Identification and validation of a locus for wheat maximum root length independent of parental reproductive environment

**DOI:** 10.3389/fpls.2022.999414

**Published:** 2022-09-12

**Authors:** Huangxin Chen, Conghao Zhao, Yaoyao Yang, Zhaoyong Zeng, Wei Li, Yanlin Liu, Huaping Tang, Qiang Xu, Mei Deng, Qiantao Jiang, Guoyue Chen, Yuanying Peng, Yunfeng Jiang, Yun Jiang, Yuming Wei, Youliang Zheng, Xiujin Lan, Jian Ma

**Affiliations:** ^1^State Key Laboratory of Crop Gene Exploration and Utilization in Southwest China, Sichuan Agricultural University, Chengdu, China; ^2^Triticeae Research Institute, Sichuan Agricultural University, Chengdu, China; ^3^Institute of Biotechnology and Nuclear Technology Research, Sichuan Academy of Agricultural Sciences, Chengdu, China

**Keywords:** maximum root length, quantitative trait loci, wheat, Wheat55K SNP array, parental reproductive environment

## Abstract

Maximum root length (MRL) plays an important role in the uptake of nutrients and resisting abiotic stresses. Understanding the genetic mechanism of root development is of great significance for genetic improvement of wheat. Previous studies have confirmed that parental reproductive environment (PRE) has a significant impact on growth and development of the next generation in the whole life cycle of a given plant. In this study, a recombinant inbred line population genotyped using the Wheat55K SNP array, was used to map quantitative trait loci (QTL) for wheat seedling MRL based on the harvested seeds from five different PREs. A total of 5 QTL located on chromosomes 3D and 7A were identified. Among them, *QMrl.sicau-2SY-3D.2* located in a 4.0 cM interval on chromosome 3D was likely independent of PREs. *QMrl.sicau-2SY-7A.2* was detected in two tests and probably influenced by PREs. The effect of *QMrl.sicau-2SY-3D.2* was further validated using the tightly linked kompetitive allele specific PCR (KASP) marker, *KASP-AX-111589572*, in populations with different genetic backgrounds. Lines with a combination of positive alleles from *QMrl.sicau-2SY-3D.2* and *QMrl.sicau-2SY-7A.2* have significantly longer MRL. Furthermore, four genes (*TraesCS3D03G0612000*, *TraesCS3D03G0608400*, *TraesCS3D03G0613600*, and *TraesCS3D03G0602400*) mainly expressed in wheat root were predicted to be associated with root growth. Taken together, this study reports on a major QTL independent of PREs and lays a foundation for understanding the regulation mechanism of wheat MRL at the seedling stage.

## Introduction

Wheat, one of the earliest domesticated cereals, is extensively grown worldwide serving as human food and livestock feed (Shewry, 2009). Wheat yield urgently awaits to be enhanced because of the growing population and rising production costs (Godfray et al., 2010). Root system architecture (RSA) plays a pivotal role in transformation and transportation of substances and determines yield potential in wheat (Ober et al., 2021). A deep RSA helps plants to resist drought stress by absorbing water and nutrients from deep soil layers (Uga et al., 2011). The difference in nitrogen uptake capacity among various wheat genotypes was mainly caused by root length (Puccio et al., 2021). Thus, an appropriate maximum root length (MRL) can improve RSA with a great potential for yield improvement in wheat breeding.

Recently, some genes or quantitative trait loci (QTL) associated with MRL were identified in wheat (Li et al., 2021a). For instance, overexpression of *TaRNAC1* increased wheat root length, biomass and drought tolerance and improved yield under water limitation (Chen et al., 2018). *TaVSR1-B* encoded a vacuolar sorting receptor protein and was associated with wheat root depth at the booting stage (Wang et al., 2021). The wheat *SHORT ROOT LENGTH 1* as an ethylene responsive factor transcription factor, controlled root length in an auxin-dependent pathway (Zhuang et al., 2021). Dwarf genes (*Rht8*, *Rht12*, and *Rht18*) in wheat have been reported to affect MRL in the field (Ingvordsen et al., 2022). Moreover, 33 QTL for four wheat root traits including seven for MRL were detected using hydroponic culture and soil-filled pot methods (Zheng et al., 2019). Nine QTL for MRL were detected under normal and salt treatments in a wheat recombinant inbred line (RIL) population. However, it is worth noting that the influence of parental reproductive environment (PRE) on root development has not been mentioned or discussed in these studies on detection of QTL for root-related traits in wheat.

Growth and development of plants can be affected by PRE in addition to the environment and heredity (Martienssen and Colot, 2001; Elwell et al., 2011). PRE affected seeds size, weight, vigor and stress tolerance of the progenies, and further impacted plant growth performance including root elongation (Grant-Downton and Dickinson, 2005; Nosalewicz et al., 2016). For example, drought priming on parents could induce thermo-tolerance in the offspring of wheat (Zhang et al., 2016). The progeny of the stressed parents maintained longer roots even under low biomass distribution at drought for spring barley (Nosalewicz et al., 2016). Progenies produced from the warm PRE showed better germination rates, root elongation growth, leaf biomass, and seed production compared with those from the cold PRE in *Arabidopsis* (BlÖDner et al., 2007). These results indicate that PRE potentially affects root growth. Considering that PRE, which is not conducive to the development of the next generation, may bring the risk of production reduction, it is necessary to identify major QTL for MRL independent of PRE in wheat.

In this study, five hydroponic tests were conducted for detecting QTL of MRL at seedling stage using seeds harvested from five different PREs in a wheat RIL population. Two major QTL, *QMrl.sicau-2SY-3D.2* (likely independent of PREs) and *QMrl.sicau-2SY-7A.2* (likely influenced by PREs) were identified. The correlations between MRL and other agronomic traits were also analyzed. The effect of these two loci on MRL were further evaluated. *QMrl.sicau-2SY-3D.2* was further validated in two populations with different genetic backgrounds. In addition, candidate genes for *QMrl.sicau-2SY-3D.2* were predicted. These results are useful for the selection of wheat lines with different MRL based on molecular markers.

## Materials and methods

### Plant materials

A previously reported RIL population (2SY) consisting of 126 F_7_ RILs from a cross between 20828 and SY95-71 was used for QTL mapping (Liu et al., 2020b). The wheat line 20828 (G214-5/3/Chuanyu19//Lang 9247/50788) with more spikelet number per spike (SNS) (Ding et al., 2022) is highly resistant to strip rust (Ma et al., 2019; Liu et al., 2020a). SY95-71 (Eronga83/Fan 6//Fan 6) is a stable line with a well-developed root system showing longer MRL (Zheng et al., 2019) and a better plant architecture (Liu et al., 2020b,^2022^). Two populations derived from the cross of HTGW3/SY95-71 (HTG3SY, F_3_, 131 lines) (Chen et al., 2022) and S849-8/SY95-71 (SSY, F_7_, 214 lines) (Qu et al., 2022) were used to verify the major QTL. The aforementioned plant materials are available from Triticeae Research Institute in Sichuan Agricultural University.

### Hydroponic culture and experimental design

MRL was measured at seedling stage in the greenhouse using hydroponic culture method as described by Ma et al. (2017). The greenhouse was maintained at 20 ± 4°C and 65%/85% (day/night) relative humidity with a 16 h/8 h day/night photoperiod. Seeds of 2SY population harvested from different PREs including Ya’an (103°0′E, 29°58′N) in 2017 and 2018, Chongzhou (103°38′E, 30°32′N) in 2018 and 2019, and Wenjiang (103°51′ E, 30°43′ N) in 2019 of Sichuan Province in China were selected (T1-T5, T for test) (Qu et al., 2021; Chen et al., 2022). Seeds of HTG3SY and SSY were harvested in Chongzhou and Wenjiang in 2021, respectively. The planting and field management strategy were carried out according to the local standard practices (Liu et al., 2020b).

In the investigated populations, 20 seeds with similar size were selected from each line. The seeds were soaked in 10% sodium hypochlorite for 5 min for surface disinfection, rinsed several times with sterile water, and placed in petri dishes with filter paper moistened with sterile water. After a week, three seedlings from each line with similar growth and development status were transferred in the plastic boxes (50 cm × 40 cm × 30 cm) filled with Hoagland nutrient solution and fixed using a sponge to keep the roots suspended. The nutrient solution was replaced weekly and the air pump was continuously used to supply oxygen to the seedlings. The whole experiment was completely randomized and repeated three times.

### Data analyses

After four weeks cultured in nutrient solution, MRL (the length from roots base to tip of the primary root, cm/plant) of seedlings was measured directly with a ruler (Ma et al., 2017). The average data of three plants for MRL was calculated for subsequent analysis. The IBM SPSS Statistics v27^1^ was used to analyze Pearson’s correlation and Student’s *t*-test (*P*< 0.05). The best linear unbiased prediction (BLUP) for target trait from different tests was calculated with SAS v8.0.^2^ Quantile-Quantile plot and boxplot were drawn with Origin Pro 2021 v9.8.^3^

In addition, the BLUP datasets of RSA-related traits, including dry root–shoot ratio (DRS), root area (RA), root diameter (RD), root dry weight (RDW), root forks (RF), root number (RN), root tips (RT), root volume (RV), shoot dry weight (SDW), and total root length (TRL) in 2SY population were used to analyze Pearson’s correlation (Chen et al., 2022). The BLUP datasets of yield-related traits, including tiller number (TN) (Liu et al., 2020b), plant height (PH) (Liu et al., 2020b), spike extension length (SEL) (Li et al., 2020), SNS (Ding et al., 2022), and thousand-grain weight (TGW) (Qu et al., 2021) in 2SY population were used to analyze Pearson’s correlation and for Student’s *t*-test in this study.

### Quantitative trait loci analysis

This study adopted the previous genetic map constructed using Wheat55K SNP Array that covers a total genetic distance of 4,273.03 cM with mean marker density of 1.69 cM/marker (Liu et al., 2020b). The inclusive composite interval mapping-additive and dominance of biparental population module (BIP) in IciMapping v4.2 (Meng et al., 2015) was used to detect QTL for MRL. The running speed, PIN value, and logarithm of odds (LOD) value of threshold were set at 1.0 cM, 0.001, 3, respectively (You et al., 2021). QTL identified in more than two tests were considered to be stably expressed.

Epistatic of IciMapping v4.2 was used for epistatic QTL (eQTL) analysis with parameters setting as follows: LOD = 5.0, PIN = 0.001, and step = 1.0 cM (Ren et al., 2021). QTL were named based on the International Rules of Genetic Nomenclature (McIntosh et al., 2013), where “Mrl,” “sicau,” and “2SY” represented “Maximum root length,” “Sichuan Agricultural University” and the mapping population, respectively.

### Comparison with reported quantitative trait loci and prediction of candidate genes

The sequences of flanking markers closely linked to the major QTL for MRL were retrieved from our previous study (Liu et al., 2020b). Those for each previously reported QTL linked to MRL were downloaded from T3/Wheat^4^ and GrainGenes 3.0.^5^ We further blasted them against the genome of “Chinese spring” (CS v2.1) (Zhu et al., 2021) on WheatOmics (Ma et al., 2021) to get their corresponding physical locations. Furthermore, candidate genes of the major QTL and their functional annotations and expression patterns (International Wheat Genome Sequencing Consortium [IWGSC], 2014) were obtained from the WheatOmics.

### Marker development and quantitative trait loci validation

A flanking marker *AX-111589572* was converted into kompetitive allele specific PCR (KASP) marker *KASP-AX-111589572* (Supplementary Table 1) to validate the genetic effects of the major QTL in different genetic backgrounds. FX96*™* Real-Time System was used for genotyping. The whole volume of 10 μL containing 0.75 μL template DNA, 1.4 μL mixture of primers, 2.85 μL deionized water, and 5 μL SsoFast EvaGreen mixture was used for the amplification reactions. PCR reaction procedure was set as follow: 15 min at 94°C, 40 cycles of 20 s at 94°C, and 60 s at 61–55°C (dropping 0.6°C per cycle).

We randomly selected 86 and 131 lines from HTG3SY and SSY populations, respectively. They were further divided into three groups based on the genotyping results: lines with homozygous alleles from SY95-71, those from HTGW3 or S849-8, and heterozygous ones. Student’s *t* test was used to evaluate the phenotypic differences between the two groups with contrary alleles in each of these two populations.

## Results

### Analysis of phenotypic data

**FIGURE 1:**
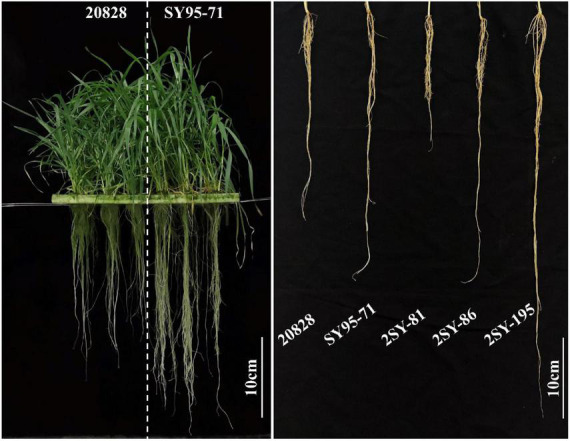
Maximum root length (MRL) of the parent 20828, SY95-71, and partial RILs. The white bar represents the scale = 10 cm.

**FIGURE 2:**
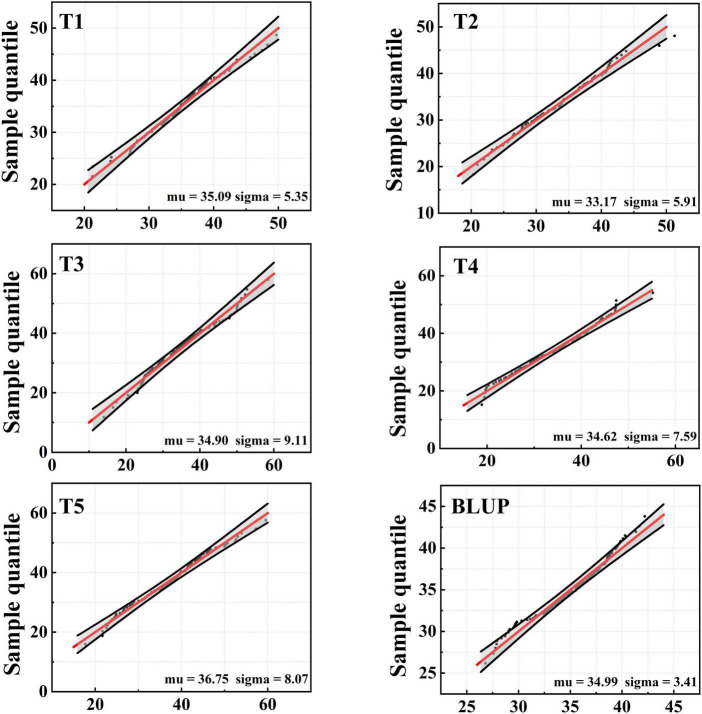
Frequency distribution of maximum root length (MRL) for five tests and with BLUP dataset.

SY95-71 had significantly longer MRL than 20828 in all tests except T3 (*P* < 0.05). The MRL of 20828 ranged from 20.33 cm to 28.60 cm, while that of SY95-71 ranged from 29.87 cm to 38.13 cm (Figure 1 and Table 1). The minimum and maximum MRL of the 2SY population were 14.00 and 59.55 cm, with mean values of 35.09, 33.17, 34.62, 34.90, and 36.75 cm in five tests, respectively (Table 1). Coefficient of variation (CV) was 15.25–26.10% for all tests. The frequency distribution was continuous and close to normal distribution in all tests, indicating that MRL was a trait of polygenic inheritance (Figure 2). In addition, significant and positive correlations for MRL were detected among five tests except TI and T4; T2 and T3 (Supplementary Table 2).

**TABLE 1:** Phenotypic variation of maximum root length (MRL) for five tests in 20828/SY95-71(2SY) population.

Tests	Parents		2SY RILs			
	20828	SY95-71	Min-max	Mean	SD	CV (%)
T1	27.23	38.13**	21.28–49.70	35.09	5.35	15.25
T2	20.33	30.10*	20.33–51.25	33.17	5.91	17.82
T3	N	29.87	18.89–55.16	34.62	7.59	21.92
T4	24.07	32.43**	14.00–58.46	34.90	9.11	26.10
T5	28.60	35.62*	17.72–59.55	36.75	8.07	21.96
BLUP	28.70	33.71	26.81–42.18	34.99	3.41	9.75

SD standard deviation, CV coefficient of variation, BLUP best linear unbiased prediction, N data missed; * and ** represent significance at the 0.05 and 0.01 level.

### Phenotypic correlation between maximum root length and other traits

Phenotypic correlations between MRL and other RSA-related traits (DRS, RA, RD, RDW, RF, RN, RT, RV, SDW, and TRL) were calculated based on the BLUP datasets (Table 2). Pearson’s correlation coefficient ranged from 0.11 to 0.54. MRL was significantly correlated with all the other RSA-related traits except RD and RN.

**TABLE 2:** Correlation coefficients between maximum root length (MRL) and root system architecture (RSA)-related traits in the 20828/SY95-71(2SY) population.

Trait	DRS	RA	RD	RDW	RF	RN	RT	RV	SDW	TRL
MRL	0.25**	0.49**	0.11	0.54**	0.20*	0.15	0.44**	0.48**	0.36**	0.49**

DRS, dry root–shoot ratio; RA, root area; RD, root diameter; RDW, root dry weight; RF, root forks; RN, root number; RT, root tips; RV, root volume; SDW, shoot dry weight; TRL, total root length. * and ** represent significance at the 0.05 and 0.01 level.

Moreover, the relationships between MRL and yield-related traits (TN, PH, SEL, SNS, and TGW) were analyzed based on the BLUP datasets (Table 3). Pearson’s correlation coefficient ranged from –0.001 to 0.28. Significant and positive correlations were detected between MRL and TN, PH, and SEL (*P* < 0.05). However, MRL was not significantly correlated with SNS and TGW.

**TABLE 3:** Correlation coefficients between maximum root length (MRL) and yield-related traits in the 20828/SY95-71(2SY) population.

Trait	TN	PH	SEL	SNS	TGW
MRL	0.20*	0.19*	0.28**	–0.15	–0.001

TN, tiller number; PH, plant height; SEL, spike extension length; SNS, spikelet number per spike; TGW, thousand-grain weight. * and ** represent significance at the 0.05 and 0.01 level.

### Quantitative trait loci mapping for maximum root length and epistatic analysis

**FIGURE 3:**
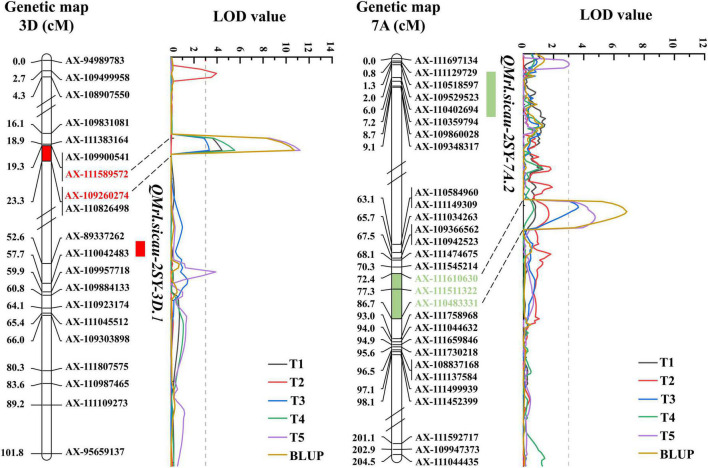
Genetic maps of the major quantitative trait loci (QTL).

Five QTL for MRL were detected and they were located on chromosomes 3D (3) and 7A (2), explaining 6.19–28.57% of phenotypic variation with LOD value ranging between 3.04 and 11.21 (Table 4). *QMrl.sicau-2SY-3D.2* as a major locus can be detected in four tests and the BLUP dataset and was mapped between *AX-111589572* and *AX-109260274* on chromosome 3D, which may not be affected by PREs (Figure 3). It explained 12.06–28.57% of phenotypic variation with LOD value ranging from 3.32 to 11.21. *QMrl.sicau-2SY-7A.2* accounted for 10.70–17.96% of phenotypic variation and was identified in T3, T5, and BLUP dataset (Figure 3). It was likely influenced by PREs. Minor QTL *QMrl.sicau-2SY-3D.1*, *QMrl.sicau-2SY-3D.3*, and *QMrl.sicau-2SY-7A.1* were detected in single test and explained 6.19–17.00% of phenotypic variation. The positive alleles of all QTL were contributed by SY95-71 except *QMrl.sicau-2SY-3D.3* (Table 4).

**TABLE 4:** Quantitative trait loci (QTL) for maximum root length (MRL) identified from five tests in the 20828/SY95-71(2SY) population.

QTL	Test	Position (cM)	Left marker	Right marker	LOD	PVE (%)	Add
*QMrl.sicau-2SY-3D.1*	T2	4	*AX-109499958*	*AX-108907550*	3.95	17.00	–2.24
*QMrl.sicau-2SY-3D.2*	T1	23	*AX-111589572*	*AX-109260274*	4.43	16.91	–2.25
	T3	23	*AX-111589572*	*AX-109260274*	3.32	12.06	–3.07
	T4	23	*AX-111589572*	*AX-109260274*	5.55	20.25	–3.22
	T5	23	*AX-111589572*	*AX-109260274*	11.21	25.10	–4.08
	BLUP	23	*AX-111589572*	*AX-109260274*	10.73	28.57	–1.68
*QMrl.sicau-2SY-3D.3*	T5	53	*AX-89337262*	*AX-110042483*	3.90	8.31	2.32
*QMrl.sicau-2SY-7A.1*	T5	5	*AX-109529523*	*AX-110402694*	3.04	6.19	–2.07
*QMrl.sicau-2SY-7A.2*	T3	76	*AX-111610630*	*AX-111511322*	3.64	14.66	–3.36
	T5	81	*AX-111511322*	*AX-110483331*	4.76	10.70	–2.65
	BLUP	78	*AX-111511322*	*AX-110483331*	6.85	17.96	–1.31

LOD, logarithm of odds; PVE, phenotype variance explained, Add additive effect of a QTL; BLUP, best linear unbiased prediction. T for Test; “sicau” means Sichuan Agricultural University; Positive value of Add indicates that alleles from 20828 are increasing the trait scores, and negative value of Add indicates that alleles from SY95-71 are increasing the trait scores.

**FIGURE 4:**
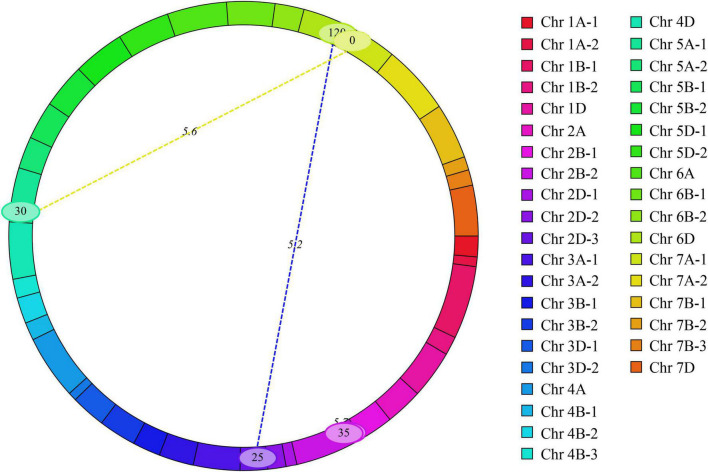
Epistatic quantitative trait loci (QTL) were detected across 21 chromosomes.

Three pairs of eQTL were detected for MRL in T2, T4, and BLUP dataset (Figure 4 and Supplementary Table 3). Their LOD and phenotypic variation values ranged from 5.23 to 5.70 and 16.56 to 22.03%, respectively. Among them, two pairs showed negatively epistatic effect value indicating that the epistatic effect of the recombinant genotype was higher than parental genotype and the other one showed positively epistatic effect value. There was no epistatic relationship between eQTL and those detected in the BIP analysis. These results suggested that these eQTL indirectly affected phenotypes through interactions.

### Evaluation of the genetic effect for the major quantitative trait loci

**FIGURE 5:**
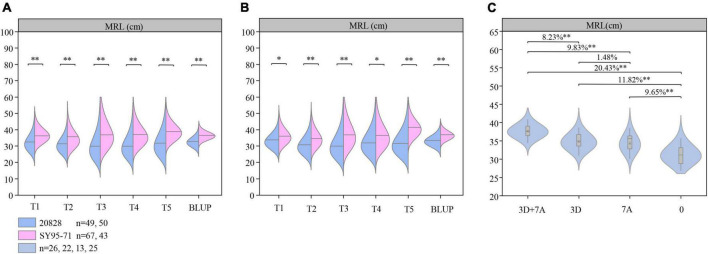
The genetic effect of major quantitative trait loci (QTL) *QMrl.sicau-2SY-3D.2* and *QMrl.sicau-2SY-7A.2*. Genetic effects of *QMrl.sicau-2SY-3D.2* and *QMrl.sicau-2SY-7A.2* in 20828/SY95-71(2SY) population **(A,B)**. The pyramiding effect of *QMrl.sicau-2SY-3D.2* and *QMrl.sicau-2SY-7A.2* on maximum root length (MRL) **(C)**. ^∗^ and ^∗∗^ represent significance at the 0.05 and 0.01 level.

Flanking markers closely linked to the two major QTL were further used to analyze their genetic effect (Figure 5). According to the genotypes of flanking markers of *QMrl.sicau-2SY-3D.2* and *QMrl.sicau-2SY-7A.2*, two groups with contrary homozygous alleles of corresponding QTL were divided in 2SY RILs, respectively (Figures 5A,B). Student’s *t*-test showed that MRL of the group carrying positive alleles of *QMrl.sicau-2SY-3D.2* or *QMrl.sicau-2SY-7A.2* were significantly longer than those carrying negative ones in five tests and BLUP dataset.

The interactions between *QMrl.sicau-2SY-3D.2* and *QMrl.sicau-2SY-7A.2* on increasing MRL were further analyzed in the 2SY RILs based on the BLUP dataset (Figure 5C). 2SY RILs were classified into four groups based on genotypes of the flanking markers. They were group A: lines with a combination of positive alleles from *QMrl.sicau-2SY-3D.2* and *QMrl.sicau-2SY-7A.2*; B: those from *QMrl.sicau-2SY-3D.2* only; C: those from *QMrl.sicau-2SY-7A.2* only; and D: those with neither *QMrl.sicau-2SY-3D.2* nor *QMrl.sicau-2SY-7A.2*. As expected, MRL of group A significantly increased by 20.33, 9.83, and 8.23% compared to group D, C, and B, respectively. MRL of groups B and C significantly increased by 11.82 and 9.65% compared to group D. There was no significant differences between group B and C.

### Validation of the major locus *QMrl.sicau-2SY-3D.2* in different genetic backgrounds

**FIGURE 6:**
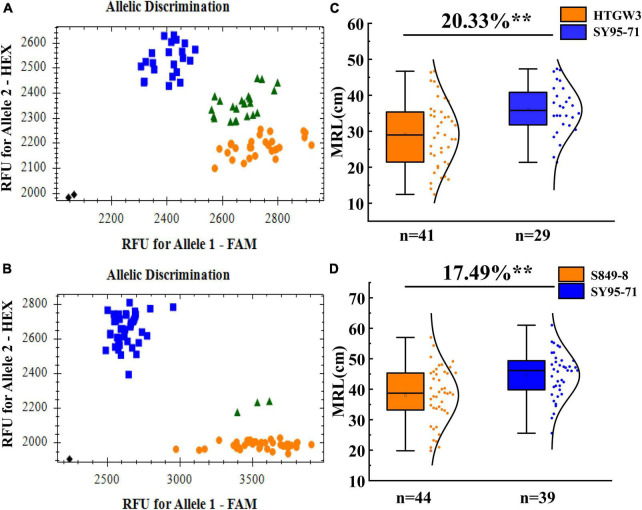
Validation of *QMrl.sicau-2SY-3D.2* in two populations with different genetic backgrounds. Blue round represents lines with the allele of SY95-71 (FAM fluorescence), orange box frame represents lines with the allele of HTGW3 **(A)** and S849-8 **(B)** (HEX fluorescence). Fluorescence PCR typing part results of the Kompetitive Allele-Specific PCR (KASP) marker *KASP*-AX-111589572 in HTG3SY **(A)** and SSY **(B)** population. Effects of *QMrl.sicau-2SY-3D.2* in two validation populations of HTG3SY **(C)** and SSY **(D)** population. ** represents significance at the 0.01 level.

The effect of the major QTL *QMrl.sicau-2SY-3D.2* was further validated in different genetic backgrounds as it was likely independent of PREs and can be stably detected (Table 4). The newly developed KASP marker (*KASP-AX-111589572*) tightly linked to *QMrl.sicau-2SY-3D.2* detected polymorphism between parent SY95-71 and HTGW3 or S849-8. Each population was divided into two groups with contrary homozygous alleles of *QMrl.sicau-2SY-3D.2* (Figures 6A,B). Lines with positive alleles of *QMrl.sicau-2SY-3D.2* from SY95-71 had significantly longer MRL than those with negative ones from other parents (Figures 6C,D). The differences in MRL between the two groups ranged from 17.49 to 20.33%.

## Discussion

### Exploration of maximum root length loci independent of parental reproductive environments

Developing a deep RSA is a vitally important strategy for well crop growth, grain yield improvement, and enhanced abiotic stress tolerance (Li et al., 2021a). For example, the gene *DEEPER ROOTING 1* increased root angle, whereby roots grew deeper facilitating high yield under drought conditions in rice (Uga et al., 2013). In addition, previous studies showed that PRE can affect growth of the next generation throughout its life cycle and may be one of the underlying triggering factors for crop yield decline (BlÖDner et al., 2007; Elwell et al., 2011; Nosalewicz et al., 2016). In this study, significant differences of CV were observed in five tests with different PREs under the same experimental conditions (Table 1). We thus speculate that PREs may affect the MRL of offspring. Compared with *QMrl.sicau-2SY-7A.2*, *QMrl.sicau-2SY-3D.2* may be a QTL independent of PREs and can be detected in most tests. The effect of PRE on MRL may be epigenetic, and changes in DNA methylation in the genome can be inherited over many generations (Kinoshita and Seki, 2014; Yang et al., 2020). The underlying epigenetic mechanisms of MRL needs to be elucidated in wheat (Kong et al., 2020). Utilization of QTL independent of PRE, like *QMrl.sicau-2SY-3D.2*, may maintain the development of MRL under various PREs and improve wheat yield in breeding.

### Relationships between maximum root length and other traits

MRL is an indication of root development potential for wheat yield improvement (Li et al., 2021a). Similar to previous studies (Li et al., 2019; Yang et al., 2021), MRL was strongly correlated with other RSA-related traits except RD and RN in this study (Table 2). By increasing the RA, RT, RV, and TRL, the contact area between roots and soil can be increased leading to the improvement of the efficiency of nutrient absorption for plants (Ryser, 2006). Ideal root depth is beneficial to root development. Meanwhile, nutrients absorbed by the roots can also be transported to the shoots (Baslam et al., 2021). MRL was positively correlated with TN, PH and SEL in this study (Table 3), showing that improvement of MRL could promote the morphological improvement of aboveground for wheat. However, no significant correlations were detected between MRL and SNS and TGW (Table 3). Xie et al. (2017) also found that there was no correlation between MRL and TGW, which might be related to the increase of grain number per spike. Lines with longer MRL likely absorb more nutrients at vegetative growth stage compared to reproductive growth stage, possibly resulting in more TN, higher PH, and longer SEL in our present study.

### Comparison of *QMrl.sicau-2SY-3D.2* and *QMrl.sicau-2SY-7A.2* to other loci

*QMrl.sicau-2SY-3D.2* was physically located between *AX-111589572* (361.45Mbp) and *AX-109260274* (368.53Mbp) on chromosome arm 3DL (Figure 3 and Table 4). Few QTL for MRL have been reported in wheat (Supplementary Table 4), especially on chromosome 3D. For example, *Qse.sau-3D* (10.64–13.28 Mbp) located on chromosome arm 3DS can affect root length (Pu et al., 2018). *QMrl.saw-1A*, *QMrl.saw-3A*, and *QMrl.saw-7D* were detected on 1A, 3A, and 7DL, respectively (Zheng et al., 2019). *QMrl-2A.2* was located between markers *Xwmc632* and *Xwmc582* closer to 202.86 Mbp on chromosome 2A (Kabir et al., 2015). *QRl-2A* and *QRl-2B.1* were identified to be linked to *AX-109366069* (64.41 Mbp) and *AX-111606522* (12.69 Mbp), respectively (Luo et al., 2021). A QTL controlling MRL was located between *AX-89595949* and *AX-111067788* at 486.96–489.10 Mbp on chromosome 7B (Fan et al., 2018). Thus, *QMrl.sicau-2SY-3D.2* was likely different from those reported previously.

Besides, *QRL.caas-7AL* was mapped between *AX-109966788* and *AX-94819074* (731.90–742.24 Mb) on chromosome arm 7AL (Yang et al., 2021), suggested that it might be allelic to *QMrl.sicau-2SY-7A.2* (652.28–669.74 Mbp).

### Genetic effect of *QMrl.sicau-2SY-3D.2* and *QMrl.sicau-2SY-7A.2*

Previous results showed that pyramiding of multiple excellent QTL can significantly improve corresponding traits (Li et al., 2020, 2021b; Ren et al., 2022). The effect of a single gene is limited, and pyramiding effect between multiple genes is not a simple accumulation due to the complex interaction mechanism (Pakeerathan et al., 2019). We further explained the relationship between *QMrl.sicau-2SY-3D.2* and *QMrl.sicau-2SY-7A.2 via* pyramiding analysis (Figure 5C). The MRL of lines with a combination of positive alleles from *QMrl.sicau-2SY-3D.2* and *QMrl.sicau-2SY-7A.2* was significantly higher than others indicating that these two loci can interact to significantly enhance MRL. There is a complex genetic relationship between the two loci which yet to be further analyzed.

In addition, three pairs of eQTL for MRL were detected (Figure 4 and Supplementary Table 3). These eQTL were not repeatedly detected in different tests and epistatic effect needs to be further explored in 2SY population.

### Potential genes in the interval of *QMrl.sicau-2SY-3D.2*

Totally, 66 high-confidence genes were annotated in the corresponding chromosomal intervals of CS v2.1 genome for *QMrl.sicau-2SY-3D.2*. Expression pattern analysis showed that 26 genes had higher expression level in roots than other organs (Supplementary Table 5). Among them, four genes (*TraesCS3D03G0612000*, *TraesCS-3D03G0608400*, *TraesCS3D03G0613600*, and *TraesCS3D03G0602400*) were mainly expressed in roots (Supplementary Figure 1). It’s worth noting that *TraesCS3D03G0612000* is an ortholog of the *Arabidopsis RACK1* gene. The *Arabidopsis* genome contains three *RACK1* paralogs, *RACK1A*, *RACK1B* and *RACK1C* (Chen et al., 2006). Previous studies have shown that the primary length of the *rack1a-1* mutant was slightly shorter than that of the wild type in *Arabidopsis* (Guo and Chen, 2008). *TraesCS3D03G0602400* encodes a GDSL esterase/lipase that was involved in seed development, lipid metabolism, abiotic stress, and pathogen defense. Transgenic rapeseed plants with independent overexpression of *AtGDSL1* and *BnGDSL1* showed that the root length of oilseed rape was longer when the basal levels of lipase activity was higher (Ding et al., 2019). *TraesCS3D03G0608400* encodes a serine/threonine-protein phosphatase. Delayed seedling establishment, longer primary roots, and higher yields were reported under normal growth conditions by overexpressing *TaSnRK2.4*, an serine/threonine protein kinase in wheat (Mao et al., 2009). *TraesCS3D03G0613600* encodes an ATP synthase subunit. Transgenic tobacco over-expressing *RMtATP6*, which encodes a subunit of ATP synthase, increased root length in salt-tolerance tests (Zhang et al., 2005). Consequently, these genes are likely involved in root elongation and may be useful for fine mapping and gene cloning for *QMrl.sicau-2SY-3D.2*.

## Conclusion

Two major QTL for MRL were detected on chromosomes 3D and 7A in this study. MRL can be significantly improved by pyramiding *QMrl.sicau-2SY-3D.2* and *QMrl.sicau-2SY-7A.2*. *QMrl.sicau-2SY-3D.2* was likely independent of PREs and it was successfully validated in two populations with different genetic backgrounds. Genetic correlations between MRL and other RSA-related and yield-related traits were also evaluated. The major QTL *QMrl.sicau-2SY-3D.2* and its linked KASP marker will be helpful in wheat breeding and gene cloning.

## Data availability statement

The original contributions presented in this study are included in the article/Supplementary material, further inquiries can be directed to the corresponding author/s.

## Author contributions

HC and CZ collaborated to complete the research and wrote the manuscript. YY, ZZ, WL, and YL helped phenotype measurement. HT and MD helped data analysis and markers development. QX, QJ, GC, and YP contributed to writing original manuscript. YFJ and YJ helped and supported the data analysis and data interpretation. YW, YZ, and XL involved in the project supervision and administration. JM was the founder of the whole experiment, directed the completion of the experiment, and conducted an in-depth review of the manuscript. All authors participated in the study and approved the final manuscript.

## Footnotes

1
http://en.wikipedia.org/wiki/SPSS

2
https://www.sas.com

3
https://www.originlab.com/

4
https://wheat.triticeaetoolbox.org/

5
https://graingenes.org/GG3/

## Supplementary material

Supplementary Figure 1 Expression pattern of *TraesCS3D03G0602400*, *TraesCS3D03G0612000*, *TraesCS3D03G0613600*, and *TraesCS-3D03G0608400* for different parts in wheat.

Supplementary Table 1 Information of KASP makers applied in this research.
